# Drug‐related problems among older adults with Mild Cognitive Impairment and dementia receiving care at Multi‐specialty Interprofessional Team‐based (MINT) memory clinic

**DOI:** 10.1002/alz.086917

**Published:** 2025-01-09

**Authors:** Rishabh Sharma, Linda Lee, Feng Chang, Tejal Patel

**Affiliations:** ^1^ University of Waterloo, Waterloo, ON Canada; ^2^ McMaster University, Kitchener, ON Canada

## Abstract

**Background:**

Up to 30% of hospitalizations in older adults living with Mild Cognitive Impairment (MCI) and dementia are attributed to drug‐related problems (DRPs), including adverse drug reactions, drug interactions, potentially inappropriate medication (PIM) use, and medication non‐adherence. This study categorizes the identified DRPs according to the Pharmaceutical Care Network Europe (PCNE) Classification for DRPs version 9.1.

**Methods:**

A cross‐sectional study was carried out with older adults receiving care for MCI or dementia at an Interdisciplinary memory clinic over eight months. The study employed four tools: the Medication Appropriateness Index (MAI), and the Medication Review in Cognitive Impairment and Dementia (MedRevCiD) checklist, Beers Criteria 2023, and Screening Tool of Older Persons Potentially Inappropriate Prescription (STOPP) 2023 to conduct a medication review. Identified DRPs were categorized according to their types and root causes with the PCNE classification system, version 9.1.

**Results:**

Forty‐four participants (45.5% female, n = 20) were enrolled. The average age of the study participants was 80.2 years (standard deviation (SD) 6.2). Most of the participants (61.4%, n = 28) had six or more comorbidities ((mean ± SD) 6.7 ± 3.4). A total of 375 medications were prescribed to the 44 study participants. The median number of medications per day was 7.5 (interquartile range (IQR): 6 per day). A total of 119 DRPs were detected in 36 patients, with an average of 2.7 DRPs per patient. The most common type of DRP was within the classification of treatment safety, accounting for 63% of the reported DRPs (n = 75), followed by treatment effectiveness (15.9%, n = 19). The most common cause of DRPs noted was inappropriate drugs according to guidelines/formulary (43.6%, n = 52), followed by an inappropriate combination of drugs, or drugs and herbal or drug‐drug interaction (21%, n = 25) from the drug selection category. A total of 53 recommendations were made by the pharmacist in the medical records for the study participants.

**Conclusion:**

The high prevalence of DRPs, notably those involving treatment safety and inappropriate drug selection, emphasizes the importance of medication reviews and tailored interventions to improve medication management and safety in this at‐risk population.

